# miR-200c Regulates *IL8* Expression by Targeting *IKBKB*: A Potential Mediator of Inflammation in Leiomyoma Pathogenesis

**DOI:** 10.1371/journal.pone.0095370

**Published:** 2014-04-22

**Authors:** Tsai-Der Chuang, Omid Khorram

**Affiliations:** Department of Obstetrics and Gynecology, University of Florida, Gainesville, Florida, United States of America; Sun Yat-sen University Medical School, China

## Abstract

We have previously reported that leiomyoma expressed lower levels of miR-200c and elevated *IL8* as compared to paired myometrium. Here we addressed the regulatory functions of miR-200c on the expression of inflammatory mediators and cellular viability using leiomyomas and paired myometrium and their isolated primary smooth muscle cells. Our results indicated that gain-of function or knockdown of miR-200c in leiomyoma smooth muscle cells (LSMC) regulated IL8 mRNA and protein expression through direct targeting of *IKBKB* and alteration of NF-kB activity. Additionally, leiomyoma expressed higher levels of phosphorylated IKBKB with no significant difference in the level of IKBKB mRNA and protein as compared to matched myometrium. Gain-of function of miR-200c in LSMC resulted in decreased IkBαphosphorylation and p65 nuclear translocation, which led to decreased p65 transcriptional activity of *IL8* promoter, and increased caspase 3/7 activity which was not reversible following IL8 restoration. Collectively, our results suggest that NF-κB signaling pathway is a target of miR-200c regulatory function, and low level of miR-200c expression in leiomyoma by transcriptional regulation of inflammatory mediators such as IL8, in part account for development of leiomyomas.

## Introduction

Uterine leiomyoma (fibroids) are benign gynecologic tumors that develop during the reproductive age and symptomatic tumors account for 1/3 of all hysterectomies performed in the United States. The factors that initiate leiomyoma’s development are unknown. Leiomyomas are composed of cells with aberrant proliferation and exhibit elevated expression of network of genes with pro-inflammatory and pro-fibrotic activities which play a central role in their growth and associated symptoms [1]–[3]. Accumulated evidence suggests that microRNAs (miRNA), a member of non-protein coding small RNA, functions as key regulator of protein coding genes expression [4], [5], and their aberrant expression has been associated with a wide range of disorders, including inflammatory and fibrotic disorders [6], [7]. Nuclear factor-κB (NF-κB) is an established key transcriptional regulator of many genes functionally associated with inflammation, fibrosis and tumorigenesis [8]–[10]. NF-κB comprises of several DNA binding proteins consisting of p50 (NF-κB 1), p52 (NF-κB 2), p65 (Rel A), Rel B and c-Rel. Functionally, NF-κB is sequestered in the cytoplasm in association with IκBs. Phosphorylation of the major IκB protein, IκBα by IκB kinase (IKKα or IKKβ) and rapid proteasome-dependent degradation results in NFκB dissociation and nuclear translocation where it binds to consensus motif of specific target genes and regulates their expression [10]. In addition, IKKβ through phosphorylation of p65 in a NF-κB-independent manner has been shown to promote apoptosis, inflammation and tumorigenesis [11]. In the uterus, the expression and nuclear localization of NF-κB p65 has been demonstrated in myometrium during parturition leading to regulation of several pro-inflammatory cytokines, including IL8 which in myometrial smooth muscle cells (MSMC) promotes premature labor [12].

Several components of NF-κB signaling pathway have been validated as direct targets of multiple miRNAs, including miR-181b, miR-199a, miR-10a and miR-155 [13]–[17]. Altered expression of miR-200 family, including miR-200c have been reported to target the expression of specific genes functionally involved in phenotypic cellular characteristic and tumorigenesis [18], [19], and to enhance pro-inflammatory responses implicated in vascular complications [20]. We have reported that leiomyomas expressed lower levels of miR-200c, and overexpression of miR-200c acting through functional regulation of ZEBs resulted in increased expression of E-cadherin causing phenotypic alteration of isolated leiomyoma smooth muscle cells (LSMC) [21]. Interestingly, NF-κB p65 subunit has been found to associate with E-cadherin and other cell-adhesion components. Repression of E-cadherin resulted in nuclear translocation of NF-κB p65 leading to transcriptional activation of mesenchymal genes such as fibronectin [22].

In this study we further explored the regulatory function of miR-200c on the expression of specific target genes whose products promote and maintain a pro-inflammatory environment which contributes to the development of leiomyomas. In cultured LSMC we found that overexpression of miR-200c altered *IL8* expression through a mechanism involving suppression of *IKBKB*, rate of IkBα phosphorylation and p65 nuclear translocation all of which contributed to altered NF-κB activity and p65 transcriptional regulation of IL8 promoter. Our results provide further evidence for several key regulatory functions of miR-200c on specific target genes that functionally promote inflammation and cell survival, events central to tissue fibrosis and tumorigenesis that are common characteristics of leiomyomas.

## Materials and Methods

### Ethics Statement

Leiomyomas and paired myometrium were collected at the University of Florida affiliated Shands Hospital and Harbor-UCLA Medical Center with prior approval obtained from the University of Florida Institutional Review Board (#283–94) and LA BioMed at Harbor-UCLA Medical Center Institutional Review Board (#036247) with exemptions for obtaining written informed consent from both committees.

### Tissue Collection and Leiomyoma and Myometrial Smooth Muscle Cell Isolation

Portions of uterine leiomyoma and matched myometrium from premenopausal women were collected from patients (n = 49), scheduled to undergo hysterectomy. The patients’ age ranged from 27 to 61 years (median = 44±7.2) and they were not taking any hormonal medications for at least three months prior to surgery. All leiomyomas used in this study were 2 to 5 cm in diameter. Tissues were snap frozen and stored in liquid nitrogen for further analysis, or used for isolation of LSMC and MSMC as previously described [23]. Only LSMC and MSMC at passages p1 to p4 were used for all experiments. All supplies for isolation and culturing of these cells were purchased from Sigma-Aldrich (St. Louis, MO), Invitrogen (Carlsbad, CA) and Fisher Scientific (Atlanta, GA).

### Gain or Loss of Function of miR-200c

LSMC were seeded at a cell density of 3.5×10^4^/well in 6-well plates and at sub-confluence transfected with 50 nM of pre-miR-200c, anti-miR-200c, pre-miR negative (preNC) or anti-miR negative control (antiNC) (Applied Biosystems, Foster city, CA) for 48 to 96 hrs using PureFection transfection reagent (System Biosciences, Inc., Mountain View, CA) according to the manufacturer’s protocol.

### RNA Isolation and Quantitative RT-PCR Analysis

Total RNA was extracted from paired tissues and cell cultures using Trizol (Invitrogen). The quantity and quality of the isolated RNAs was determined (ND-1000 Spectrophotometer, NanoDrop Technologies, Wilmington, DE) and 10 ng (for miRNA) or 2 µg was reverse transcribed using specific stem-loop primer for miR-200c or random primers for *IL8* and *IKBKB* according to the manufacturer’s guidelines (Applied Biosystems). Quantitative RT-PCR was carried out using TaqMan or SYBR gene expression master mix, TaqMan miRNA or TaqMan gene expression assays (Applied Biosystems). Reactions were incubated for 10 min at 95°C followed by 40 cycles of 15 seconds at 95°C and 1 min at 60°C and level of mRNAs and miRNAs expression was determined using Applied Biosystems 7300 Detection System with 18S and RNU6B used for normalization, respectively. All reactions were run in triplicate and relative expression was analyzed with the comparative cycle threshold method (2^−ΔΔ^CT) according to the manufacturer (Applied Biosystems). Values were expressed as fold changes compared to control group. The primers sequences used in SYBR system for amplification of IL8 and 18S were sense, 5′-ACCTTTCCACCCCAAATTTATCA-3′; antisense, 5′-TTTCTGTGTTGGCGCAGTGT-3′ and sense, 5′-GACGGACCAGAGCGAAAGC-3′; antisense, 5′-CCTCCGACTTTCGTTCTTGATT-3′, respectively.

### Immunoblotting

Total protein isolated from LSMC transfected with 50 nM pre-miR-200c, anti-miR-200c, pre-miR negative (preNC) or anti-miR negative control (antiNC) for 48 hrs was subjected to immunoblotting as previously described [24]. Antibodies against c-Jun (Santa Cruz Biotechnology, Santa Cruz, CA), E-cadherin, IKBKB, p-IKBKB (Ser 177/181), IkBα, p-IkBα (Ser 32/36), p65, Calnexin and Calpain (Cell Signaling Technology, Inc., Danvers, MA) were used to detect specific protein expression. The membranes were also stripped and probed with α-tubulin (Abcam, Inc., Cambridge, MA) or GAPDH antibody (Santa Cruz Biotechnology) serving as loading control. The band densities were determined using image J program (http://imagej.nih.gov/ij/), normalized to α-tubulin or GAPDH, and expressed as a ratios relative to the control group designated as 1.

### Immunoprecipitation

For immunoprecipitation, cells were harvested and lysed in ice-cold cell lysis buffer (20 mM Tris-HCl, pH 7.5, 150 mM NaCl, 1 mM EDTA, 1 mM EGTA, 1% Nonidet P-40, plus phosphatase and protease inhibitors). An aliquot of 300 µg of lysate protein was incubated with anti-p65 or anti-E-cadherin (Cell Signaling Technology) primary antibody at 4°C overnight. Protein A/G-agarose beads (Merck, Darmstadt, Germany) were then added for an additional 3 hrs at 4°C. The immunocomplexes were spun at 700×*g* for 1 min, washed three times with ice-cold lysis buffer, and suspended in SDS-PAGE sample buffer.

### ELISA

The IL8 content in culture media was determined using Quantikine human CXCL8/IL8 ELISA kits (R&D Systems, Minneapolis, MN) as previously described [25]. The levels of IL8 were calculated as pg/mg of protein and reported as fold change compared to control experiments.

### Luciferase Reporter Assays

LSMC were seeded in six-well plates until reaching sub-confluence and transiently cotransfected with 50 nM pre-miR-200c oligonucleotides or pre-miR negative control (preNC) and a luciferase reporter plasmid (1 µg/well) containing NF-kB conserved binding sequences for NF-kB activity detection (Signosis, Sunnyvale, CA), IL-8 (gift from Dr. R.G., Pestell [26]), IKBKB (gift from Dr. Gil Mor [15]) and pRL-TK plasmid (Promega, Madison, WI) encoding Renilla luciferase (0.2 µg/well) as a control for differences in transfection efficiency using PureFection transfection reagent. Firefly and Renilla luciferase activities were measured after 48 hrs of transfection using the Dual-Luciferase Reporter Assay System (Promega). Firefly luciferase activity was normalized to Renilla luciferase activity and the level of induction was reported as the mean ± SEM of three experiments performed in duplicates and compared with a ratio in cells transfected with preNC independently set at 1.

### Sub-fractionation of Cellular Proteins

LSMC were transfected with 50 nM pre-miR-200c or preNC for 48 hrs. Cell membrane, cytoplasmic, and nuclear proteins were fractionated following the protocol of the subcellular protein fractionation kit (Thermal Scientific, Waltham, MA).

### Immunofluorescence Microscopy

LSMC were transfected with 50 nM preNC or pre-miR-200c for 48 hrs, or treated with IL-1b (5 ng/ml) for 1 h as positive control. Cells were fixed for 15 min in 4% paraformaldehyde and followed by permeabilization with 0.2% Triton X-100 for 10 min. After blocking with 2% bovine serum albumin, the cells were incubated with rabbit anti-p65 (Cell Signaling Technology) antibody following by FITC-conjugated goat secondary antibody (Santa Cruz Biotechnology). Cell nuclei were also stained with diamidino-2-phenylindole hydrochloride (DAPI) for 5 min at 37°C. After mounting slides using antifade reagent (Invitrogen), visualization was carried out using laser spinning confocal microscopy (TCS SP2; Leica, Wetzlar, Germany) with the accompanying software (Slidebook, Irving, TX).

### Chromatin Immunoprecipitation

CHIP analysis was carried out using Simple CHIP enzymatic chromatin IP kit (Cell Signaling) according to the manufacturer’s protocol. PCR-amplification was performed using primers designed to amplify p65 binding site on IL8 promoter [27] (forward: 5′- AAGAAAACTTTCGTCATACTCCG -3′, reverse: 5′- TGGCTTTTTATATCATCACCCTAC -3′), under PCR condition of 95°C for 15 min, 33–35 cycles of 94°C for 20 s, 60°C for 20 s, and 72°C for 20 s. The Ct values were normalized to corresponding inputs and percent input method was used to quantitate the values of the immunoprecipitated DNA.

### Caspase 3/7 Activity Assay

MSMC and LSMC isolated from the same paired tissues were seeded at 1000 cells/well in 96-well plates and cultured for 48 hrs. The cells were then transfected with preNC or pre-miR-200c as described above and caspase 3/7 activity was determined after 96 hrs incubation using Apo-One homogeneous caspase 3/7 assay (Promega) according to the manufacturer’s protocol. The rate of caspase 3/7 activity was determined by measuring fluorescence using multi-plate reader (Molecular Device, Inc., Sunnyvale, CA) at excitation 485 nm and emission 527 nm.

### Statistical Analysis

Whenever appropriate, the results were reported as mean ± SEM. All *in vitro* experiments were performed using primary cells prepared from at least three different patients. The Student’s t-test was used for comparisons involving two groups. For comparisons among multiple groups, analysis of variance (ANOVA) followed by a Tukey’s HSD post hoc pairwise multiple comparison was used. Statistical significance was established at P<0.05.

## Results

### Gain-of Function of miR-200c Leads to Down-regulation of IL8

**Figure 1:**
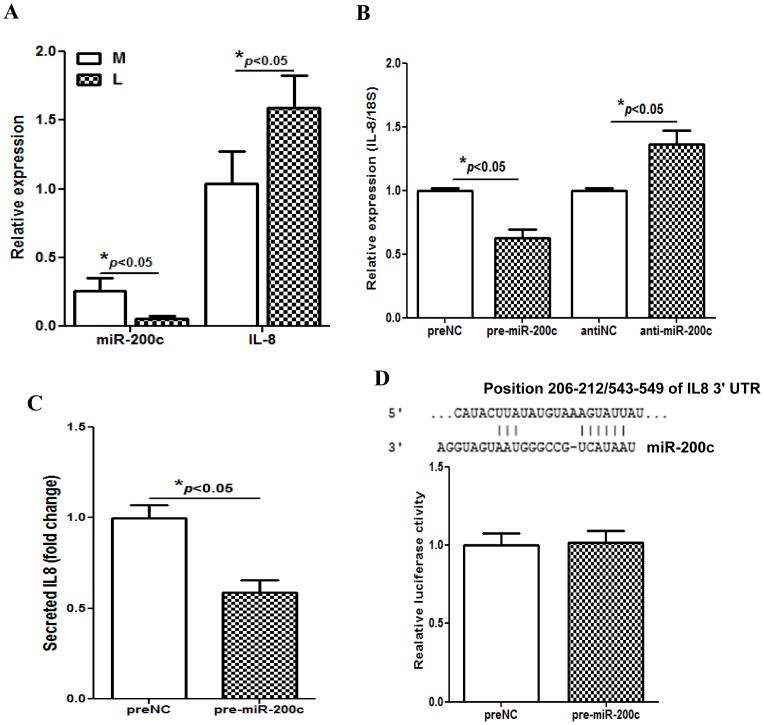
Gain-of function of miR-200c led to down-regulation of IL8. Bar graphs in figure 1A show the relative expression of miR-200c and *IL8* in leiomyoma (L) and matched myometrium (M) (N = 49). Relative expression of IL8 mRNA (Fig. 1B) and IL8 content (Fig. 1C) determined by QRT-PCR and ELISA of culture conditioned media of LSMC transfected with pre-miR-200c, anti-miR-200c or negative control (preNC or antiNC) for 48 hrs and 72 hrs respectively. Figure 1D shows relative luciferase activity in LSMC co-transfected with pGL3 construct carrying a 3′UTR fragment of *IL8*, firefly luciferase reporters, pRL-TK and pre-miR-200c or preNC. The ratio of firefly:Renilla was determined and reported as relative luciferase activity as compared to preNC. Sequence alignment of miR-200c seed regions and IL8 mRNAs target sites at their 3′UTRs with the coordinated positions are shown at the top of graph. The data are reported as mean ± SEM of experiments performed using 3 to 5 sets of isolated LSMC prepared from leiomyoma from three different patients. The results were analyzed using non-parametric student t-test and corresponding lines with asterisks on the bars denote statistical significance.

We have previously reported that leiomyomas expressed lower levels of miR-200c with concurrent elevated IL8 expression as compared to paired myometrium [21], [25]. Here we confirmed these findings and found that the relative expression of miR-200c and *IL8* display an inverse relationship in leiomyomas and myometrium (Figure 1A). Using isolated LSMC cells as an *in vitro* model we found that gain-of function of miR-200c resulted in a significant suppression of IL8 mRNA (Figure 1B) and protein (Figure 1C) levels. In contrast miR-200c knockdown in these cells produced the opposite effect with a significant increase in *IL8* mRNA (Figure 1B). Since miRNAs through complementary interaction with the sequences in 3′UTR of their target genes regulate their expression, we assessed if miR-200c through interaction with *IL8* 3′UTR regulates *IL8* expression. As shown in Fig. 1D, luciferase reporter assay indicated that miR-200c has limited or no interaction with *IL8* 3′UTR, suggesting that the regulatory function of miR-200c on IL8 expression, at least in LSMC, occurs through other mechanism(s).

### Gain-of Function of miR-200c Suppressed NF-kB Pathway through Targeting IKBKB

**Figure 2:**
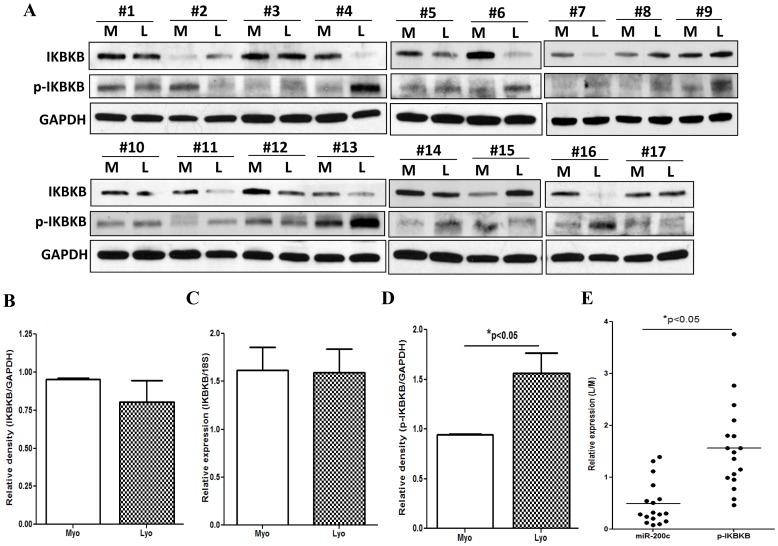
phosphorylated IKBKB displayed an inverse relationship with miR-200c expression. Figure 2A shows western blot analysis of IKBKB and phosphorylated IKBKB (p-IKBKB) (Ser 177/181) of tissue extracts (N = 17) from leiomyomas (L) and paired myometrium (M) with GAPDH was used as loading control. The relative band densities of IKBKB (Fig. 2B) and phosphorylated IKBKB (Fig. 2D) in leiomyoma and paired myometrium were determined and compared with values in myometrium independently set as 1 for each pair. Figure 2C shows relative expression of IKBKB mRNA level in leiomyoma and paired myometrium from untreated group (N = 49). The data were analyzed using nonparametric student t-test. Figure 2E shows the relative expression miR-200c as compared to the level of phosphorylated IKBKB (p-IKBKB) in above 17 leiomyomas as compared to matched myometrium. The results were analyzed using paired student t-test and corresponding lines with asterisks denote statistical significance.

To further address the potential regulatory function of miR-200c on IL8 expression in LSMC, we selected *IKBKB* which serves as an upstream regulator of *IL8* and predicted as a target of miR-200c (TargetScan http://www.targetscan.org). Using leiomyomas and matched myometrium we first analyzed and found that IKBKB mRNA and protein are expressed in all paired tissues examined with considerable variation in their levels and no significant difference in their mean expression values (Figs. 2A, 2B and 2C). However, the level of phosphorylated IKBKB (Ser 177/181) was significantly higher in leiomyomas as compared with matched myometrium (p<0.05, Fig. 2A and 2D) and displayed an inverse relationship with miR-200c expression among the same pairs of tissues (Fig. 2E).

**Figure 3:**
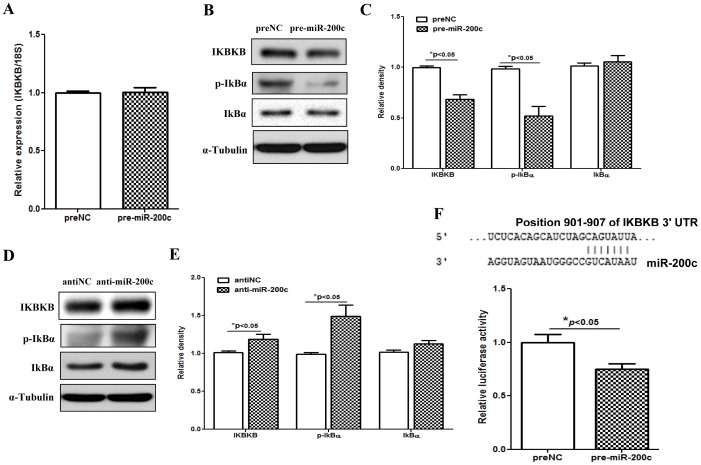
IKBKB is a direct target of miR-200c. Figure 3A shows the influence of gain-of function of miR-200c in LSMC on the expression of IKBKB after 48 hrs transfection as determined by QRT-PCR. Figures B, C, D and E show western blots and band intensity analysis of IKBKB, IkBα and phosphorylated IkBα (p-IkBα) (Ser 32/36) in LSMC following transfection with preNC, pre-miR-200c, antiNC or anti-miR-200c for 48 hrs with α-tubulin used as loading control. The relative band intensities were determined and the values for preNC or antiNC were independently set as 1 for comparative analysis. Figure 3F shows luciferase reporter assay following co-transfection of LSMC with firefly luciferase reporter carrying a 3′UTR fragment of IKBKB, pRL-TK, and pre-miR-200c or preNC. The ratio of Firefly:Renilla was determined after 48 hrs and reported as relative luciferase activity as compared to preNC independently set as 1 for each assessment. The results presented as mean ± SEM of three sets of independent experiments using LSMC isolated from 3 patients and analyzed using unpaired student t test. Asterisks denote statistical significance indicated by corresponding lines. Sequence alignment of miR-200c seed regions and IKBKB mRNAs target sites at their 3′UTRs with the coordinated positions are shown at the top of graph.

**Figure 4:**
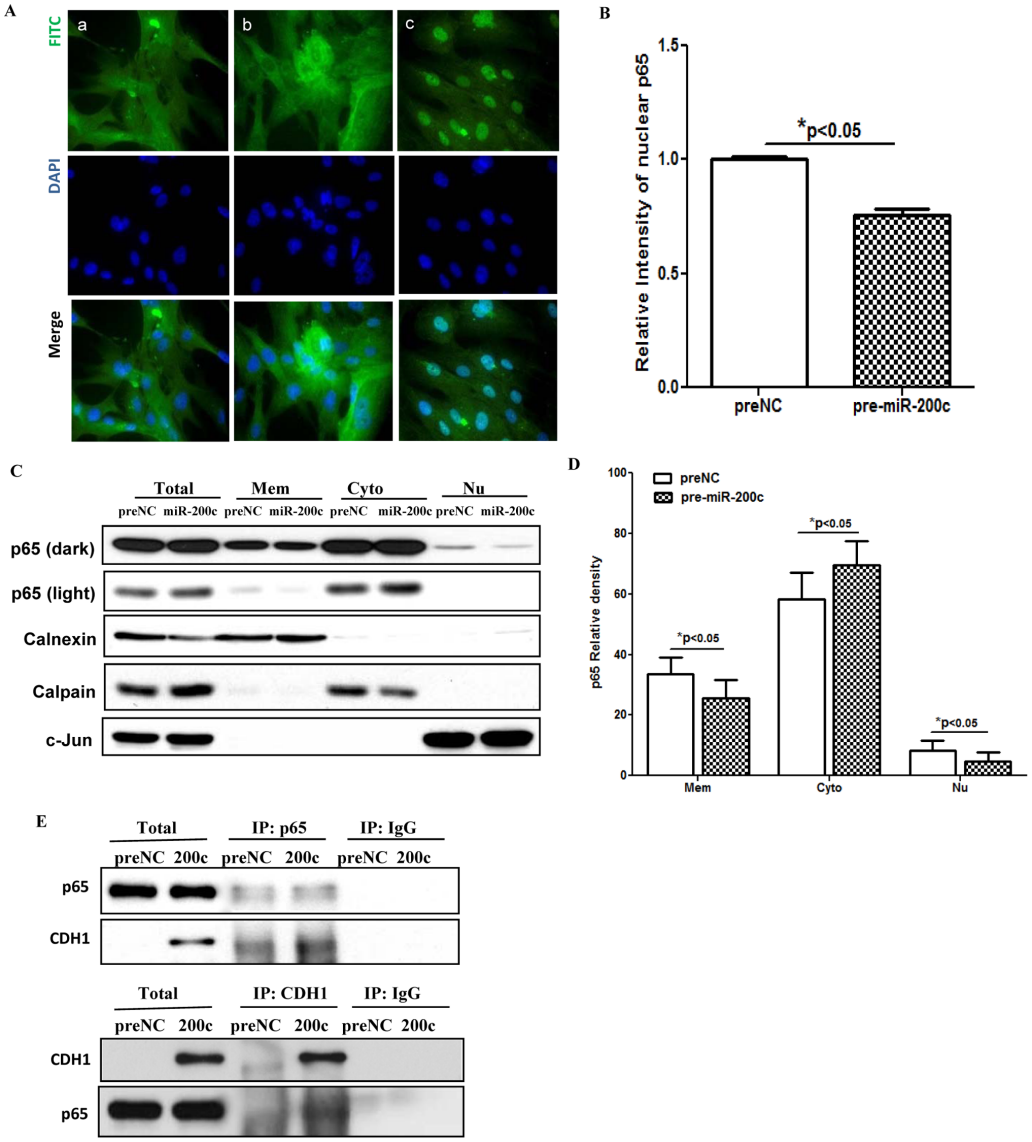
Gain-of function of miR-200c suppressed NF-kB signaling pathway. Figure 4A shows immunofluorescence staining of LSMC transfected with pre-NC, (panel a), pre-miR-200c (panel b) for 48 hrs, or treated with IL-1b (5 ng/ml) for 1 h as positive control (panel c). The cells were fixed and immunostained for p65 subunit of NF-kB (green, p65) using p65 antibody and counter-stained with DAPI (blue, nucleus) with arrows indicating to NF-kB p65 nuclear immunostaining. Figure B shows mean ± SEM of relative nuclear immunostaining intensity of p65 (*p<0.05 compared to preNC). Figure 4C shows immunoblot analysis of NF-kB p65 in LSMC (3×10^5^/100 mm dish) transfected with pre-miR-200c or preNC for 48 hrs. The cells were harvested and subfractionated into membrane (Mem), cytoplasmic (Cyto), and nuclear (Nu) proteins and subjected to immunoblot analysis, with Calnexin, Calpain and c-Jun served as markers for the respective subcellular fractions. The NF-kB p65 band intensity (Fig. D) was semiquantified and reported as mean ± SEM of percentage of total p65 associated with Mem, Cyto and Nu fractions in cells transfected with pre-miR-200c or preNC (*p<0.05 when compared to preNC). Figure 4E shows immunoprecipitation and immunoblotting of LSMC total cell lysates after transfection with pre-miR-200c or preNC using p65 or CDH1 (E-cadherin) antibodies. Similar results were obtained from three sets of independent experiments.

**Figure 5:**
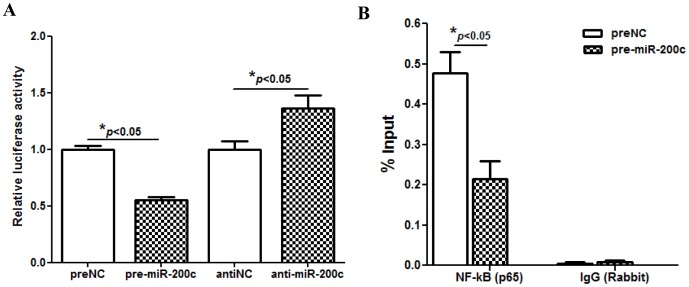
Gain-of function of miR-200c inhibited NF-kB p65 binding activity in IL8 promoter. Figure 5A shows the level of NF-kB activity in LSMC transfected with a luciferase reporter construct containing preserved NF-kB binding sites, pRL-TK, pre-miR-200c, anti-miR-200c, preNC or antiNC. The ratio of Firefly:Renilla was determined after 48 hrs and reported as relative luciferase activity as compared to preNC which was independently set as 1 for each cell. (Fig. 5B) NF-kB binding ability in endogenous IL8 promoter accessed by CHIP assay. LSMC were transfected with pre-miR-200c or preNC. After incubation for 48 hrs, cells were harvested and processed for CHIP assay using p65 antibody. Immunoprecipitated chromatin was analyzed by PCR using the specific primer for IL8 promoter and presented by percent input method. The results are presented as mean ± SEM of three sets of independent experiments using primary LSMC isolated from 3 patients and analyzed using non-parametric student unpaired t test with asterisks denote statistical significance indicated by corresponding lines.

Gain-of function of miR-200c in LSMC had a limited effect on IKBKB expression at mRNA level, but significantly repressed IKBKB at translational level which was increased following miR-200c knockdown (Fig. 3A to 3E). The regulatory function of miR-200c on IKBKB expression occurred through a direct interaction with 3′UTR of IKBKB (Fig. 3F). Since phosphorylation of IκBα by IκB kinases [IKKα (IKBKA) or IKKβ (IKBKB)], and rapid proteasome-dependent degradation, results in NF-κB dissociation and nuclear translocation, where NF-κB binds to consensus motif of specific target genes and regulates their expression, we examined the expression of IkBα and assessed the level of phosphorylated IkBα at serine 32/36 following gain- and loss-of function of miR-200c. As illustrated gain-of function of miR-200c repressed, while knockdown of miR-200c increased the level of phosphorylated IkBα in LSMC (Fig. 3B to 3E). Furthermore, immunofluorescence and subcellular fractionation analysis indicated that a significant portion of p65 was present in the LSMC cytoplasmic fraction and gain-of function of miR-200c reduced the level of p65 nucleus translocation (Fig. 4A to 4D). Since elevated expression of E-cadherin has been shown to result in cytoplasmic sequestration of NF-κB p65 subunit [28], we found that gain-of function of miR-200c in LSMC which enhanced E-cadherin expression [21], also resulted in cytoplasmic sequestration of p65 (Fig. 4E). Additionally, as indicated by the luciferase reporter assay, gain-of function of miR-200c reduced NF-kB activity, while it was induced following knockdown of miR-200c in LSMC (Fig. 5A). We also confirmed that gain-of function of miR-200c inhibited NF-kB p65 binding activity in *IL8* promoter as demonstrated by CHIP assay (Fig. 5B), thus resulting in suppression of IL8 mRNA and protein expression (Fig. 1B and 1 C).

### Gain-of Function of miR-200c Induces Caspase 3/7 Activity but the Effect could not be Reversed by IL8 Restoration

**Figure 6:**
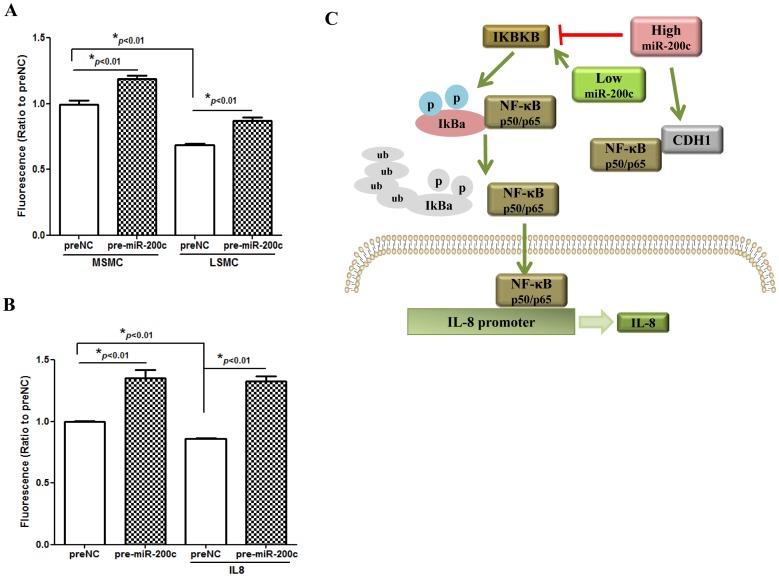
Gain-of function of miR-200c induced caspase 3/7 activity, but the effect could not be reversed by IL8 restoration. Figure 6A shows influence of gain-of function of miR-200c and preNC on caspase 3/7 activity in MSMC and LSMC after 96 hrs of incubation with culture media changed every two days. The results are reported as mean ± SEM of experiments performed in six replicates from three independent cell preparations using the same paired of primary MSMC and LSMC isolated from 3 patients and analyzed using nonparametric student t-test with asterisks denote statistical significance as compared to preNC. Figure 6B shows caspase 3/7 activity in LSMC transfected with pre-miR-200c or preNC treated with or without IL8 (50 ng/ml) for 96 hrs. The results are reported as mean ± SEM of experiments performed in six replicates, using three independent cell preparations and analyzed using nonparametric student t-test with asterisks denote statistical significance as compared to preNC. Figure 6C shows a schematic representation of aberrant expression and regulatory function of miR-200c on the expression of IL8 through NF-kB signaling pathway involving IKBKB in LSMC. Our results suggest that aberrant expression of miR-200c through functional regulation of specific target genes serve as mediator of inflammatory and tissue turnover play a key role in leiomyoma development, growth and associated symptoms.

We have also reported that gain-of function of miR-200c in LSMC resulted in cellular phenotypic transformation from a mesenchymal into an epithelial-like characteristic and decreased rate of cell proliferation [21]. Here we showed that gain-of function of miR-200c also induced caspase 3/7 activities which was significantly lower in LSMC as compared to MSMC (Fig. 6A) and coincided with lower miR-200c expression in leiomyomas (Fig. 1A). Since *IL8* has been reported to have an anti-apoptotic effect in endometrial stromal cells [29], we investigated if IL8 restoration could compensate for the effect of miR-200c on caspase 3/7 activity. Although IL8 (50 ng/ml) treatment alone reduced caspase 3/7 activity, it did not have a significant effect on miR-200c-induced caspase 3/7 activation in LSMC (Fig. 6B).

## Discussion

Several pro-inflammatory mediators, ischemia/hypoxic conditions and ovarian steroids through NF-kB signaling pathway and hormone response element regulate the expression of many genes, including pro-inflammatory and cell cycle associated genes [10], [30], [31]. The product of these genes regulate diverse cellular processes, including cell proliferation, apoptosis, migration, angiogenesis and tissue remodeling [10], [31], events that play key roles in leiomyoma’s pathogenesis. In the present study, we demonstrated that in LSMC miR-200c regulates IL8 expression which occurred indirectly through downregulation of NF-kB signaling pathway by targeting IKBKB 3′UTR. Functionally, gain-of-function of miR-200c leading to repression of IL8 in LSMC is in line with lower miR-200c and elevated IL8 expression in leiomyoma as compared to myometrium [21], [25]. In support of our observations, a recent report provided further evidence for an inverse correlation between IL8 expression and miR-200c in a large panel of cell lines [32]. NF-kB signaling pathway regulates the expression of diverse target genes, including *CCND1*, *VEGF*, ECM related genes, and proteases such as *MMP9* [33]. Collectively, the results of this part of our study suggest that aberrant expression of miR-200c, at least through its regulatory effect on NF-kB signaling pathway and specific downstream target genes contribute to the outcome of leiomyoma’s pathogenesis (Fig. 6C).

Further insight into regulatory function of NF-kB signaling pathway in leiomyoma revealed that the level of phosphorylated IKBKB at Ser-177/181 was elevated and displayed an inverse relationship with miR-200c as compared to myometrium. IKBKB is well established to account for nearly all catalytic kinase activity of the IKK holoenzyme toward IκBα, and phosphorylation at Ser-177/181 activates IKBKB [34]. As such, an elevated level of IKBKB phosphorylation and lower miR-200c expression in leiomyoma could promote an environment necessary for activation of NF-kB signaling pathway and regulation of specific target genes. Although our *in vitro* data clearly identified IKBKB as a direct target of miR-200c in LSMC, regulatory function of other miRNAs or other gene products may also target IKBKB in leiomyoma. In addition to miR-200c, several other miRNAs have been predicted to target IKBKB and *IL8* expression, of which miR-199a and miR-218 were validated to regulate *IL8* through IKBKB [15], [17], [35], and miR-17/20, miR-93/106b, miR-146a/b and miR-155 directly targeted *IL8* expression or IL8-mediated inflammation, senescence and cellular invasion [13], [25], [27], [36]. In support of our observations, other reports have demonstrated the presence of NF-kB p50 and p65 proteins in leiomyoma cells [37] and elevated IL8 mRNA level in leiomyoma with stronger immunoreactive IL8 in the edge of leiomyoma adjacent to myometrium [38], [39]. As such, an increase in NF-kB dependent- regulation of inflammatory-related genes expression, including IL8, further support the potential role of inflammatory events in the pathogenesis of leiomyomas [1]. We extended the biological significance of NF-kB signaling pathway in leiomyoma and found that miR-200c decreased IkBα phosphorylation which caused NF-kB p65 cytoplasmic sequestration and through this pathway repressed IL8 expression in LSMC. The direct consequence of reduced IκBα activity and degradation which result in p65/p50 heterodimer retention in the cytoplasm, often lead to decreased NF-κB transcriptional activity [40]. Although IkBα is regulated through phosphorylation and proteasome-mediated degradation, we found that miR-200c knockdown increased IkBα phosphorylation in LSMC. In support of our findings with miR-200c, knockdown of miR-10a has also been reported to increase IkBα phosphorylation and nuclear retention of NF-kB in human aortic endothelial cells (HAEC) resulting in elevated production of inflammatory biomarkers, such as monocyte chemotactic protein 1 (*MCP-1*), *IL6*, *IL8*, vascular cell adhesion molecule 1 (*VCAM-1*), and E-selectin [16]. However, miR-10a regulatory actions on these genes were indirect and occurred through repression of mitogen-activated protein kinase kinase kinase 7 (*MAP3K7*) and β-transducin repeat-containing gene (*βTRC*) which promotes proteasomal degradation of IκBα and p65 nuclear translocation [16]. Since NF-kB signaling pathway regulates IkBα expression under a dynamic balance between co-activators and co-repressors [41], the significance of miR-200c regulatory functions, specifically on IkBα and wide spectrum of biological processes regulated by NF-κB signaling pathway could have a direct impact on pathogenesis of leiomyoma.

Recent reports have indicated that IL8 inhibits cell apoptosis through the regulation of anti-apoptotic gene expression, including increased ratios of Bcl-xL:Bcl-xS and Bcl-2:Bax leading to enhanced cell survival [42], [43]. Additionally, IL8 decreased sensitivity of tumor cells to caspase 8-mediated, TRAIL-induced apoptosis via increased expression of c-FLIP_L_ and c-FLIP_S_ which are two isoforms of the endogenous caspase 8 inhibitor [43]. In endometrial stromal cells IL8 has also been shown to up-regulate Fas ligand (*FasL*) expression which promoted cytotoxic T lymphocytes apoptosis, a mechanism considered to serve as mediator of local immunotolerance in endometriosis [29]. Here we found that treatment of LSMC with IL8 resulted in decreased caspase 3/7 activity; however, the apoptotic effect induced by gain-of-function of miR-200c in LSMC was not reversed by IL8 treatment. Although gain-of function of miR-200c repressed IL8 expression, the results suggest that miR-200c may target other genes which encode for proteins with anti-apoptotic activities. miR-200c has been reported to reduce sensitivity of CD95-mediated apoptosis in tumor cells by targeting Fas-associated phosphatase-1 (*FAP-1*) [44], suppress migration and anoikis resistance by targeting moesin (*MSN*), fibronectin 1 (*FN1*) and neurotrophic tyrosine receptor kinase type 2 (*TrkB*) [45] and negatively regulate EGF-driven cell proliferation and motility by targeting phospholipase C gamma 1 (*PLCG1*) [46]. We have previously reported that *ZEB1* and *ZEB2* were direct targets of miR-200c in MSMC and LSMC and using microarray profiling also identified many apoptosis and motility related genes targeted by gain-of function of miR-200c in these cells [21]. The miR-200c repression of ZEBs in LSMC also resulted in a significant increase in E-cadherin expression, which is expressed at a very low level in leiomyomas and LSMC [21]. Our finding of E-cadherin interaction with NF-kB p65 subunit leading to p65 cytoplasmic sequestration is of particular interest, considering the requirement of p65 nuclear translocation for NF-kB transcriptional regulation of target genes, such as interaction with IL8 promoter. Consistent with our finding in LSMC, interaction of catalytic IKK subunits with Smad3 during TGFβ-SMAD-mediated EMT has been reported to occur independent of NF-κB activity, contributing to tumor-promoting function of TGFβ-SMAD signaling pathway and cellular invasion [47]. Specifically, Snail1 and NF-kB p65 subunit interaction with fibronectin promoter and their nuclear interactions were required for fibronectin transcription and other genes involved in cell movement [22].

In summary, we showed that leiomyoma express an elevated level of phosphorylated IKBKB at Ser-177/181 as compared to myometrium and using isolated MSMC and LSMC as an *in vitro* model provided further evidence for the regulatory function of miR-200c on IKBKB and caspase 3/7 activity. Since the NF-kB signaling pathway regulates various genes whose products regulate diverse groups of cellular activities, including cell cycle progression, inflammatory response, angiogenesis and tissue turnover, their alterations as a result of aberrant expression of miR-200c or other miRNAs with multiple target genes may play a central role in the pathogenesis of leiomyomas. Although leiomyomas development and subsequent growth is multi-factorial, our data provide strong support for unique regulatory functions for miR-200c in leiomyoma pathogenesis and associated symptoms.
